# Effect of HDL composition and particle size on the resistance of HDL to the oxidation

**DOI:** 10.1186/1476-511X-9-104

**Published:** 2010-09-23

**Authors:** Nakanishi Shuhei, Sanni Söderlund, Matti Jauhiainen, Marja-Riitta Taskinen

**Affiliations:** 1 Division of Cardiology, Department of Medicine, University of Helsinki, Helsinki, Finland; 2 National Institute for Health and Welfare, Public Health Genomics Research Unit, and FIMM, Institute for Molecular Medicine Finland, Biomedicum, Helsinki, Finland

## Abstract

**Objectives:**

To study the resistance of HDL particles to direct oxidation in respect to the distribution of HDL particles.

**Design and Methods:**

We studied HDL composition, subclass distribution, and the kinetics of CuSO_4_-induced oxidation of total HDL and HDL_3 _in vitro in 36 low-HDL-C subjects and in 41 control subjects with normal HDL-C.

**Results:**

The resistance of HDL_3 _to oxidation, as assessed from the propagation rate was significantly higher than that of total HDL. The propagation rate and diene formation during HDL oxidation in vitro was attenuated in HDL derived from low-HDL-C subjects. Propagation rate and maximal diene formation during total HDL oxidation correlated significantly with HDL mean particle size. The propagation rate of total HDL oxidation in vitro displayed a significant positive association with HDL_2 _particle mass and HDL mean particle size by multiple regression analyses.

**Conclusions:**

These observations highlight that the distribution of HDL subpopulations has important implications for the potential of HDL as an anti-oxidant source.

## Introduction

A strong body of epidemiological evidence associates low levels of high density lipoprotein cholesterol (HDL-C) with the cardiovascular disease risk [1,2]. Importantly, it was just recently reported that HDL protects against cardiovascular disease in both males and females, independent on subject's age and at all levels of risk [3].

HDL protects against atherosclerosis by several mechanisms although the reverse cholesterol transport plays the major role. HDL exerts anti-atherogenic actions also via its inherent anti-oxidative and anti-inflammatory properties [4], because it carries enzymes such as paraoxonase 1 (PON1), which may protect against oxidation as well as inflammation [4-6]. Recently it was also demonstrated that apoA-I and HDL can neutralize procoagulant properties of anionic phospholipids and thereby prevent inappropriate stimulation of blood coagulation [7]. HDL acts as an effective scavenger of superoxide anions [8], and thus it can protect low density lipoprotein (LDL) against oxidative modification in vitro [5,6,9,10]. The interplay between LDL oxidation and atherogenesis may provide additional opportunities for functional metrics of HDL. Regarding the atheroprotection it is of importance to notify that HDL particles are highly heterogeneous in their size, structure, metabolism, and biological function. Emerging evidence suggests that small, dense HDL_3_-subspecies possess a higher capacity to protect LDL against oxidation than large, light HDL_2 _particles [9-11]. Taken together, this protective action of HDL particles is of utmost importance since oxidized LDL is considered to be the most atherogenic species among the modified LDLs [12].

Subjects with low HDL-C display marked changes in their HDL composition and subclass distribution. Previous studies indicate that larger HDL_2 _particles as well as HDL mean particle size are reduced in subjects with low HDL-C [13-16]. Notably, HDL particle size correlates positively with HDL-C levels [15,17]. In addition, familial low-HDL-C subjects exhibit higher levels of inflammation markers such as high-sensitivity C-reactive protein (hsCRP) [18,19].

Low-HDL-C status is a typical complex disorder caused by interaction between several genes and environmental factors. It is a highly prevalent condition among people with type 2 diabetes and is one of the seminal features of the metabolic syndrome [20,21]. Notably, low HDL-C is the most common familial lipoprotein abnormality in subjects with premature coronary heart disease (CHD) [22-24]. Considering the global epidemics of type 2 diabetes and metabolic syndrome, the burden of low HDL-C as a risk factor of cardiovascular disease is likely to increase rapidly in the near future [25,26].

A growing body of evidence suggests that the capacity of HDL to protect LDL against oxidation is compromised in low-HDL-C subjects as compared to individuals with normal HDL-C levels [13]. In contrast, it is still unclear whether functional deficiency of HDL particles with respect to direct oxidation of its lipids is linked to any specific subclass or composition of HDL particles. This lead us to explore the oxidation susceptibility of both total HDL and HDL_3 _particles among Finnish low-HDL-C subjects, known to have clear-cut differences in HDL subspecies, particle compositions, and HDL mean particle size as compared to subjects with normal HDL-C levels.

## Subjects and methods

### Study subjects

We recruited 36 low-HDL-C subjects and 41 healthy control subjects with normal HDL-C levels for this study. Low HDL-C was defined according to the Adult Treatment Panel III (ATP III) of the National Cholesterol Education Panel [21], (HDL-C < 1.03 mmol/L in men and < 1.29 mmol/L in women). Of the 36 low-HDL-C subjects, 23 were family members from 10 low-HDL-C families, as defined previously [27]. In addition, we included, in the low-HDL-C group, 4 spouses from low-HDL-C families and 9 family members with low HDL-C from families, which did not fulfill the criteria for low-HDL-C families [14]. The control group consisted of 16 spouses from low-HDL-C and familial combined hyperlipidemia (FCHL) families, and 25 normolipidemic subjects from families, which did not fulfill the criteria for familial dyslipidemias. The control subjects were healthy with normal HDL-C levels as defined by ATPIII and did not use any lipid medication. Subjects with diabetes mellitus or estrogen therapy were excluded. Of the low-HDL-C women, 2 were postmenopausal and of the women in the control group, 5 were postmenopausal. Each participant filled out a standard questionnaire on medication, drinking, and smoking habits. The smoking status of the subjects was categorized in three groups, current, ex-, and non-smokers. Each study subject gave a written informed consent before participating in the study. All samples were collected in accordance with the Helsinki declaration, and the Ethics Committee of the Helsinki University Central Hospital approved the protocol.

### Biochemical analyses

Venous blood samples were drawn after an overnight fast for biochemical analyses. Serum and EDTA plasma were separated by low-speed centrifugation and stored at -80°C until analysis. Serum total cholesterol (TC) and triglycerides (TG) were determined with an automated Cobas Mira analyser (Hoffman-La Roche, Basel, Switzerland) by fully enzymatic methods (Hoffman-La Roche kits #0722138 and #0715166, respectively). Serum HDL-C was quantified after the precipitation of apoB-containing lipoproteins with phosphotungstic acid/magnesium chloride (Hoffman-La Roche kit #0720674). Serum LDL cholesterol was calculated from the Friedewald formula [LDL cholesterol = TC-(HDL-C)-TG/2.2] [28]. Notably TG levels of all subjects were under 4.5 mmol/l. Concentrations of apolipoprotein A-I (apoA-I), apolipoprotein A-II (apoA-II) and apolipoprotein B-100 (apoB-100) were measured by immunoturbidometric methods (for apoA-I; Wako Chemicals GmbH, Neuss, Germany, for apoA-II; Wako Chemicals GmbH and own polyclonal antibody produced in rabbits against human apoA-II, and for apoB; Orion Diagnostica, Espoo, Finland). Plasma glucose concentration was analysed by glucose dehydrogenase method (Precision-G Blood Glucose Testing System, Medisense, Abbott, Illinois, USA). The level of hsCRP was determined by a commercial kit (Konelab kit #981798, Thermo Electron Corporation, Vantaa, Finland). Subjects with hsCRP levels over 10 mg/L were excluded from the calculation. Serum PON1 activities were determined as described [29] using paraoxon as a substrate.

### Distribution and composition of HDL subfractions

HDL subclasses were isolated by ultracentrifugation, and HDL composition was analyzed as described previously [30]. Particle mass concentrations of the isolated HDL_2 _and HDL_3 _were calculated as the sum of concentrations (in mg/dl) of TG, free cholesterol, cholesteryl esters (CE), phospholipids (PL) and total protein of particles. HDL subclass distribution and HDL mean particle size were determined with native gradient gel electrophoresis as previously described [31] with minor modifications. Briefly, electrophoresis was performed by using the d ≤ 1.21 kg/l ultracentrifugally isolated lipoprotein fraction from plasma. We used Hoefer miniVE vertical electrophoresis system (Amersham Biosciences, San Fransisco, CA, USA) with native 4-22% polyacrylamide gradient gels (10×10.5 cm, PAA:BIS 19:1) prepared in the laboratory. Samples were electrophoresed at 125 V for 24 hours at +4°C in a running buffer (90 mM TRIS, 80 mM boric acid, and 3 mM EDTA, pH 8.53). Gels were stained for one hour with 0.04% Coomassie blue G-250 and destained overnight with 5% acetic acid. Gels were densitometrically scanned with Kodak digital science 1D system (Eastman Kodak Company, Rochester, NY, USA) and analyzed with ImageMaster™ 1D software (version 4.00, Amersham Pharmacia Biotech, Newcastle, UK).

We used High Molecular Weight (H.M.W.) calibration kit from Pharmacia for standardization. The characteristic molecular size intervals for HDL subclasses 2b, 2a, 3a, 3b, and 3c were used [31], and for each subpopulation, the relative area under the densitometric scan is reported. Mean HDL particle size, as the surrogate marker of the distribution of HDL subclasses, was calculated by multiplying the mean size of each HDL subclass by its relative area under the densitometric scan [32].

### Antioxidative potential of HDL subfractions

After ultracentrifugation EDTA was immediately removed from the HDL and HDL_3 _using size exclusion chromatography (PD-25 column). The pure HDL or HDL_3 _(100 μg of total HDL or HDL_3 _protein/ml, respectively) was incubated in the presence of 5 μmol/l (final concentration) of freshly prepared CuSO4 in a total volume of 0.2 ml at +27°C [33,34]. Conjugated diene formation was kinetically monitored at 234 nm in temperature-controlled spectrophotometer (Safire V2.00, Tecan Austria GmbH, Austria) every 2 minutes until 200 minutes. Every sample was run in at least six times and finally the mean value of the measurements was calculated. During the kinetics of diene accumulation, three consecutive phases were identified, the lag, the propagation, and the decomposition phase [9,13,33-35]. For each curve, the duration of lag phase, propagation rate and amount of dienes formed at the end of the propagation phase (maximal dienes) were calculated as the surrogate markers of antioxidative capacity of HDL and HDL_3_. The lag time (min) was defined as the time from the addition of CuSO4 until the intersection of this tangent with the baseline [36]. The propagation rate is defined as the slope of the tangent of the propagation phase and is expressed as nmol dienes formed/min per mg of total HDL or HDL_3 _protein. The maximum amount of conjugated dienes formation, expressed as nmol dienes formed/mg of total HDL or HDL_3 _protein, was determined as the height of maximum absorbance above baseline.

### Statistical Analysis

Statistical comparisons of clinical and biochemical parameters were performed with SAS ver.8.02 (SAS Institute Inc.). Results are expressed as means ± SD for continuous variables and as frequencies or percentages for categorical variables. Before the analyses, continuous variables with skewed distribution were log_10 _transformed, but the values in text, tables and figures are presented as nontransformed original data. Continuous variables were compared between the groups by general linear model, analysis of covariance (ANCOVA). Sex was coded as men = 1, women = 0. Smoking status was coded as current = 2, past = 1, and never = 0. Comparison of three oxidation markers between HDL_3 _and total HDL was done using the Wilcoxon rank-sum test. *P *< 0.05 was considered significant (two-tailed). The frequency distribution of the categorical variables was compared between the groups with the Chi-square test. The relationships of biochemical and clinical characteristics were examined by Pearson's correlation and Spearman correlation analysis, as appropriate. Also, multiple stepwise backward regression analyses were performed to investigate associations of propagation rate and maximal dienes of oxidation as dependent variables. Independent variables were removed from the model until the best-fitting model with the maximum adjusted multiple R2 was achieved. ApoA-II and HDL-C were not included in the same model, because these were highly correlated to apoA-I. For the same reason, LDL-C and apoB, or CE/TG ratio of HDL_2 _and HDL_2 _particle mass concentrations, or CE/TG ratio of HDL_3 _and HDL_3 _particle mass concentrations were not included in the same model.

## Results

### Clinical characteristics of the study subjects

The clinical characteristics of the study subjects are presented in Table 1. Twelve of the low-HDL-C subjects had CHD. The percentage of current smokers was slightly higher among the low-HDL-C subjects. After adjustment for age, sex, and smoking status, the low-HDL-C subjects had significantly higher BMI, waist, TG, and hsCRP. As expected and based on the selection criteria TC, apoA-I and A-II levels were reduced in the low-HDL-C subjects. However, PON1 activity was not different between the two groups.

**Table 1:** Clinical characteristics of the study subjects according to the criteria of ATPIII

	Low-HDL-C	Control	*P*
N (Men/Women)	36 (25/11)	41 (19/22)	0.027
Current smoking (%)	14 (40.0)	9 (22.0)	0.189
Coronary heart disease (%)	12 (34.3)	0 (0)	
Age (years)	43.7 ± 13.7	45.6 ± 12.7	0.802
Systolic blood pressure (mmHg)	129 ± 18	128 ± 16	0.607
Diastolic blood pressure (mmHg)	80 ± 11	81 ± 9	0.985
Body mass index (kg/m^2^)	26.2 (23.9 - 29.0)	23.7 (21.4 - 26.5)	0.001
Waist circumference (cm)	96.0 ± 10.8	84.7 ± 10.7	0.001
TC (mmol/l)	4.22 (3.69 - 5.17)	5.04 (4.31 - 5.60)	0.003
TG (mmol/l)	1.22 (1.02 - 1.62)	0.85 (0.72 - 1.08)	< 0.0001
LDL-C (pmol/l)	2.68 (2.15 - 3.53)	2.79 (2.33 - 3.29)	0.316
HDL-C (pmol/l)	0.93 (0.81 - 0.99)	1.62 (1.35 - 1.91)	< 0.0001
ApoA-I (mg/dl)	110 (104 - 117)	143 (129 - 159)	< 0.0001
ApoA-II (mg/dl)	35 (29 - 40)	37 (32 - 40)	0.035
ApoB (mg/dl)	96 (83 - 111)	88 (78 - 106)	0.657
PON1 activity (nmol/min/ml)	155 (106-211)	133 (92-265)	0.614
hsCRP (mg/l)	1.03 (0.51 - 2.91)	0.46 (0.24 - 1.71)	0.007

Data are expressed as means ± SD or median (lower and upper quartile). The two *P *values from the top are evaluated using χ2 tests. Other data are adjusted for age, sex, and smoking status.

The protein/lipid composition and the distributions of HDL subspecies in the study subjects are presented in Table 2. After adjustment for age, sex, and smoking status, the particle mass concentrations and CE/TG ratio of both HDL_2 _and HDL_3 _were markedly lower in the low-HDL-C subjects than those in control subjects. The HDL_2 _particles of the low-HDL-C subjects contained relatively more TG and total protein but less CE and phospholipids than those of the control subjects. Similarly, HDL_3 _from the low-HDL-C subjects contained relatively more TG but less CE than HDL_3 _in the control group. The proportion of HDL_2b _particles was significantly lower, whereas that of HDL_3a _and HDL_3b _were significantly higher in the low-HDL-C subjects than in the control subjects. HDL mean particle size was markedly smaller in the low-HDL-C subjects as compared to that in the control subjects.

**Table 2:** Composition (weight %) and distribution (%) of HDL derived from the low-HDL-C and control subjects

	Low-HDL-C	Control	*P*
HDL_2 _particle mass (mg/dl)	68 (58 - 90)	153 (106 - 213)	< 0.0001
HDL_2 _CE/TG ratio	3.17 (2.64 - 4.22)	6.00 (4.48 - 7.34)	< 0.0001
HDL_2 _TG (% of HDL_2_)	5.6 (4.2 - 6.6)	3.4 (2.8 - 4.5)	< 0.0001
HDL_2 _CE (% of HDL_2_)	18.9 (16.2 - 20.6)	20.8 (19.2 - 22.3)	0.002
HDL_2 _free cholesterol (% of HDL_2_)	4.7 (4.2 - 6.0)	5.0 (4.6 - 5.6)	0.573
HDL_2 _phospholipids (% of HDL_2_)	24.0 (22.0 - 26.0)	27.9 (24.8 - 29.5)	0.001
HDL_2 _protein (% of HDL_2_)	46.3 (43.1 - 49.4)	42.5 (39.9 - 46.2)	0.0007
			
HDL_3 _particle mass (mg/dl)	209 (191 -226)	242 (214 - 266)	0.0001
HDL_3 _CE/TG ratio	4.64 (3.48 - 5.96)	7.07 (5.61 - 8.80)	< 0.0001
HDL_3 _TG (% of HDL_3_)	3.7 (3.0 - 4.3)	2.5 (2.1 - 3.1)	< 0.0001
HDL_3 _CE (% of HDL_3_)	16.8 (14.9 - 18.8)	17.5 (16.5 - 19.1)	0.039
HDL_3 _free cholesterol (% of HDL_3_)	2.3 (2.0 - 2.5)	2.4 (2.2 - 2.6)	0.848
HDL_3 _phospholipids (% of HDL_3_)	22.2 (20.2 - 23.3)	22.3 (21.0 - 23.2)	0.815
HDL_3 _protein (% of HDL_3_)	54.9 (51.6 - 58.1)	55.6 (51.7 - 57.7)	0.984
			
HDL_2b _(%)	15.1 ± 5.0	30.1 ± 10.4	< 0.0001
HDL_2a _(%)	27.2 ± 5.2	26.3 ± 7.0	0.763
HDL_3a _(%)	34.9 ± 4.4	27.7 ± 6.4	< 0.0001
HDL_3b _(%)	18.5 ± 5.4	10.9 ± 4.5	< 0.0001
HDL_3c _(%)	5.3 ± 2.7	4.1 ± 3.0	0.422
HDL mean particle size (nm)	8.98 ± 0.21	9.44 ± 0.35	< 0.0001

Data are expressed as means ± SD or median (lower and upper quartile). TG: triglycerides, CE: cholesteryl esters. Statistical significances were adjusted for age, sex, and smoking status. HDL subclasses were isolated by ultracentrifugation and distribution of HDL subclasses as well as particle size were analyzed by native gradient gel electrophoresis.

### The resistance of total HDL and HDL_3 _particles to the in vitro oxidation

Both the propagation rate and maximal diene formation during total HDL oxidation were significantly higher than those observed during HDL_3 _oxidation in both control subjects and low-HDL-C subjects. The lag time, however, did not differ between HDL_3 _and total HDL oxidation in either control or low-HDL-C subjects (Table 3).

**Table 3:** Comparison of the parameters of HDL oxidation between HDL particles derived from the low-HDL-C and control subjects

		Low-HDL-C	Control	*P *value
Lag time (minute)				
	Total HDL oxidation	66.47	69.19	0.326
		(56.35 - 96.54)	(56.16 - 78.50)	
	HDL_3 _oxidation	69.40	64.04	0.206
		(55.75 - 96.51)	(54.44 - 82.45)	
	*P *value	0.427	0.176	
Propagation rate (nmol/mgHDL or HDL_3_/min)				
	Total HDL oxidation	1.242	1.543	< 0.0001
		(1.097 - 1.362)	(1.431 - 1.795)	
	HDL_3 _oxidation	1.032	1.022	0.533
		(0.924 - 1.123)	(0.807 - 1.159)	
	*P *value	< 0.0001	< 0.0001	
Maximal dienes (nmol/mgHDL or HDL_3_)				
	Total HDL oxidation	86.67	107.47	0.002
		(76.38 - 103.31)	(97.62 - 114.87)	
	HDL_3 _oxidation	71.47	71.57	0.646
		(63.26 - 78.45)	(60.89 - 84.22)	
	*P *value	< 0.0001	< 0.0001	

Data are expressed as median (lower and upper quartile). Statistical significances were adjusted for age, sex, and smoking status between low HDL and control subjects.

After adjustment for age, sex, and smoking status, propagation rate and maximal diene formation during oxidation of total HDL derived from the low-HDL-C subjects were significantly lower than the corresponding values with HDL isolated from the control group (Table 3). The lag time values of total HDL oxidation, however, were not different between the two groups. Even after further adjustment for TC, apoA-I, apoA-II, TG, BMI, waist, or hsCRP, the differences in the propagation rate of total HDL between the two groups remained significant (data not shown). In contrast, all the surrogate markers of HDL_3 _oxidation were comparable between the two groups (Table 3).

### The impact of HDL composition, particle size and systemic inflammation on the resistance of HDL to in vitro oxidation

**Figure 1:**
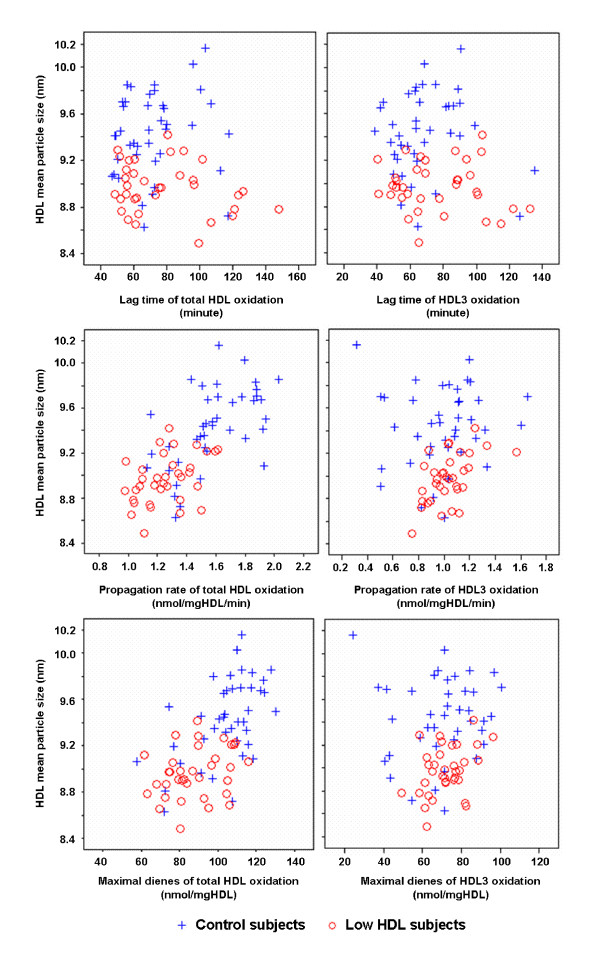
**Relationships between HDL mean particle size and oxidation parameters of total HDL and HDL_3 _derived from the low-HDL-C and control subjects**. Upper panels: relationship between HDL mean particle size and lag time; Middle panels: relationship between HDL mean particle size and propagation rates; Lower panels; relationship between HDL mean particle size and maximal diene formation.

Relationships between the three surrogate markers of HDL oxidation in vitro and HDL mean particle size are presented in Figure 1. HDL mean particle size showed strong positive correlation with the propagation rate and the maximal dienes formed during total HDL oxidation (r = 0.689, P < 0.0001, and r = 0.598, P < 0.0001, respectively). As certain plots of the control and low-HDL-C subjects overlap we analyzed the relationships in a pooled study sample. When the groups were analyzed separately HDL mean particle size correlated significantly in the control subjects with the propagation rate (r = 0.557, P = 0.0002), and with the maximal dienes formed during total HDL oxidation (r = 0.531, P = 0.0004). Similarly, the low-HDL-C subjects showed a strong positive correlation between HDL mean particle size and the propagation rate (r = 0.407, P = 0.016), but the correlation with the maximal diene formation did not reach statistical significance (r = 0.281, P = 0.103).

During oxidation of the HDL_3 _particles, a relationship existed between HDL mean particle size and the propagation rate among low HDL subjects (r = 0.451, P = 0.007). However, among control subjects or in the pooled study sample, no relationships existed between HDL mean particle size and the propagation rate or the maximal diene levels during oxidation of the HDL_3 _particles. In addition, the lag time values of total HDL or HDL_3 _during Cu-mediated oxidation did not correlate significantly with HDL mean particle size.

Finally, we performed stepwise regression analyses to determine the relationships between different variables and the markers of total HDL (Table 4). The propagation rate of total HDL oxidation was significantly associated with HDL_2 _particle mass, HDL mean particle size, and hsCRP. The maximal diene formation in total HDL oxidation was significantly associated with HDL mean particle size. In contrast, the propagation rate during HDL_3 _oxidation was significantly associated only with hsCRP. The maximal diene formation during HDL_3 _oxidation had no association with any of the measured variables.

**Table 4:** Stepwise regression analyses of the parameters of total HDL and HDL_3 _oxidation in the combined population of low HDL and control subjects

		Propagation rate		Maximal dienes	
		β	*P*	β	*P*
Total HDL oxidation					
	HDL_2 _particle mass (mg/dl)	0.390	0.005	NE	-
	HDL mean particle size (nm)	0.325	0.021	0.582	< 0.001
	hsCRP (mg/l)	-0.205	0.005	NE	-
HDL_3 _oxidation					
	hsCRP (mg/l)	-0.321	0.021	NE	-

BMI and TG were included. β: Standardized regression coefficient. NE: Factor that did not enter the final model. The adjusted multiple R2 of the regression model of propagation rate and maximal dienes of total HDL oxidation was 0.556 and 0.321, respectively. The adjusted multiple R2 of the regression model of propagation rate and maximal dienes of HDL_3 _oxidation was 0.062 and -0.008, respectively.

## Discussion

Here we report the susceptibility of total HDL and HDL_3 _particles to Cu-mediated oxidation in vitro. HDL particles were isolated from the well-characterized Finnish low-HDL-C subjects and from the healthy subjects with normal HDL-C concentrations. Previous studies have explored the effect of HDL to protect LDL from oxidation but data on the susceptibility of HDL itself to oxidation are sparse. To the best of our knowledge, this is the first study to explore the impact of HDL composition, size, and hsCRP as a surrogate marker of systemic inflammation, on direct oxidation of HDL particles in vitro using widely used copper oxidation method of Esterbauer [33] that initiates free radical reactions within HDL particles. Unexpectedly, we found that the propagation rate and diene formation during HDL oxidation in vitro was attenuated in the low-HDL-C subjects. Also, we demonstrated that the resistance of HDL_3 _particles to oxidation is higher than that of total pool of HDL particles. This suggests that HDL_3 _particles are less prone to oxidation than HDL_2 _in vitro. In addition, the resistance of HDL particles to oxidation is affected by HDL lipid/apolipoprotein composition, HDL-associated proteins other than apolipoproteins, subclass distribution, and systemic inflammation.

Interestingly, HDL particles are major carriers of lipid peroxides in circulation [36]. In fact, HDL_3 _particles possess higher PON1 activity [9] as compared with HDL_2 _particles. PON1 is considered to be a major protein conferring anti-oxidant activity of HDL [6,37,38]. Accordingly, HDL_3 _particles were more resistant than total HDL particles to direct Cu-oxidation based on the difference in the propagation rate in both groups, because HDL includes both HDL_2 _and HDL_3_. In addition, compared to HDL_3 _particles, the increase in maximal diene formation of total HDL particles was apparent in both groups. These results support previous data indicating that small, dense HDL particles have more capacity to protect LDL against oxidation than large HDL particles [9-11]. A lower propagation rate and less diene formation are commonly considered to be an indicator of increased antioxidant capacity [33,34]. In line with this it has been reported that large, less-dense HDL subspecies and high HDL concentrations tended to enhance more diene production than small, dense HDL particles [9,34,35]. In this context it is important to notify that small, dense HDL_3 _particles seem to have compromised anti-oxidative activity in subjects with type 2 diabetes and the metabolic syndrome [39,40]. The present study groups did not contain any subjects with diabetes. However, the low-HDL-C subjects had features of the metabolic syndrome such as elevated waist circumference and inflammation.

The fact that the resistance of total HDL particles to oxidation in vitro from the control subjects was significantly lower than that from the low-HDL-C subjects is a paradox. This apparent contradiction could be explained by the dramatic difference of HDL_2 _concentrations between the two groups. The concentrations of HDL_2 _particles in the purified HDL fraction from the control subjects are expected to be high compared to HDL from the low-HDL-C subjects at the same level of HDL protein since variability in HDL-C levels mainly reflects changes in the large HDL particles [41]. Accordingly, after adjustment for the ratio of HDL_2 _and HDL_3 _protein, the difference of maximal diene formation during total HDL oxidation between the two groups disappeared (data not given). Notably, the propagation rate of total HDL oxidation was inversely correlated with HDL_2 _particle mass and HDL particle size. Nevertheless, the susceptibility of HDL_3 _to oxidation in the low-HDL-C subjects was not different from that seen in HDL_3 _derived from the control subjects. This result may be explained by the fact that PON1 activity, affecting HDL_3 _anti-oxidant activity [9], was not different between the two groups in our study.

A recent paper using hypochlorous acid (HOCL) as a potent oxidant demonstrated that structural and physiochemical differences between HDL_2 _and HDL_3 _subclasses per se did not affect their intrinsic susceptibility to oxidative attack by HOCL. Instead it was dependent on the concentration (particle number or mass) employed [42]. Human HDL proteome studies have revealed that HDL-associated proteins are distributed in specific patterns across HDL subpopulations, and the noticed potent anti-oxidative activity of HDL_3 _particles is characterized by a proteome of distinct composition [43]. Taken together this part, HDL_2 _is more readily oxidized than HDL_3_, most likely because of its higher content of oxidable lipids and lower content of potentially protective proteins.

As expected in the low-HDL-C subjects, the lipid core compositions of HDL_2 _and HDL_3 _displayed clear changes such as enrichment of TG and depletion of CE. Importantly, HDL_2 _was also depleted in phospholipids and the amount of apolipoproteins relative to phospholipids was increased in the low-HDL-C subjects. These changes in physicochemical properties of HDL particles may modify the resistance of HDL subfractions to oxidation. Recently, phospholipid depletion has been shown to decrease the size of reconstituted HDL particles [44]. Therefore, the low susceptibility of HDL to oxidation in the low-HDL-C subjects may be due to the compositional changes specifically in the HDL_2 _subclass.

A short lag time during oxidation is considered to reflect enhanced susceptibility to oxidation. In our study, there were no differences in lag time of HDL_3 _and total HDL particles between the two groups. Our data are in line with other observations that the impairment of HDL to protect LDL against in vitro oxidation using HDL derived from subjects with low-HDL-C or metabolic syndrome was observed only in those oxidation parameters displayed after the lag phase [13,40]. Although the detailed mechanisms responsible for the antioxidative properties inherent for HDL particles are not well understood, both copper and HDL concentrations used in the experiments in vitro may modulate the oxidation capacity of HDL subfractions [9,35,45]. HDL particles are easily oxidized by several mechanisms including myeloperoxidase production that may be critical in vivo to produce dysfunctional HDL.

There are certain limitations in the present study and therefore extrapolations of our findings should be made with appropriate caution. It is clear that there are differences in oxidation kinetics depending on the oxidation reagent used. The antioxidant properties of HDL are modified by the availability of several HDL associated molecules such as α-tocopherol, LCAT, and estrogen [46-49] that were not measured in this study. Unfortunately we were not able to measure directly the oxidation of HDL_2 _particles in vitro due to a lack of material. As twelve patients with CHD were included in the low-HDL subjects a possibility exists that some medication such as atorvastatin [50,51] might modify the antioxidative capacity of HDL particles. However, the results when examined via excluding these twelve subjects did not differ significantly (data not shown).

The small sample size including both genders in the same analyses is recognized as a limitation of this study. However, we aimed to minimize the confounding effect of mixed gender population by gender adjustment in the statistical analyses. The recruitment of the study subjects from families may affect the results but the possible confounding factor was considered relative as the mean family size was small (2).

Although recruited from the database of familial dyslipidemias, the low-HDL-C subjects display additional features of metabolic syndrome such as elevated waist circumference and hsCRP. This is in line with our previous finding on familial low-HDL-C subjects [18]. As an isolated low-HDL-C state is rare, the cardiovascular burden of low HDL-C will focus on the subjects with the features of metabolic syndrome.

In summary, HDL lipid and protein distributions affect the antioxidative capacity of HDL_2 _and HDL_3 _as well as systemic inflammation of HDL subfractions.

## Competing interests

The authors declare that they have no competing interests.

## Authors' contributions

NS carried out the analysis of distribution and composition of HDL subfractions, oxidation assays. SS made all the statistical analyses and participated in manuscript drafting and selection of study subjects. MJ made the biochemical analyses, participated in HDL subclass analysis and study design and drafted the manuscript. MRT organized the subject material, participated in study design and drafted the manuscript. All authors read and approved the final manuscript.
